# A Never-Ending Journey in Search for Novel Cell Biology Techniques

**DOI:** 10.3390/cells11091393

**Published:** 2022-04-20

**Authors:** Alexander E. Kalyuzhny

**Affiliations:** Department of Neuroscience, University of Minnesota Twin Cities, 6-145 Jackson Hall, 321 Church St SE, Minneapolis, MN 55455, USA; kalyu001@umn.edu

Cell techniques undergo rapid advancement across different areas of biomedical research. This is dictated by the complexity of modern trends demanding novel techniques that are in many cases not just improvements and iterations of existing protocols but rather totally new approaches never utilized before. Our Special Issue presents a collection of advanced cell techniques that can be used to solve the vast majority of research tasks, and it appears that they can be adapted for diagnostic applications as well.

Seahorse analysis of cancer metabolism using 3D spheroids is a challenging task, and Campioni et al. developed an optimized protocol allowing the production of homogeneous spheroids for characterization of the metabolic activity of tumors using Seahorse technology [1].

An interesting novel protocol has been developed by Born-Torrijos et al. to isolate myxozoans from fish blood [2]. The isolation of myxozoans, the smallest obligately parasitic cnidarian animals, allows the analysis of their DNA and proteins. This is critical for investigating the mechanisms of their infiltration into the host animals and pathology, which, in turn, can help with identifying effective strategies to control myxozoan diseases.

There is a growing interest in analyzing the morphology and movements of intracellular organelles to better understand their role in normal physiology and pathology, and our volume includes two state-of-the-art techniques on this subject. The first one by Polev et al. [3] focuses on using wavelet transform, a sophisticated mathematical frequency analysis approach. Wavelet analysis resembles Fourier transform that allows the identification of the frequency of various signals but, in addition, can also determine the presence of particular frequencies at a certain point in time. Wavelet analysis was successfully applied to analyze the mobility of lysosomes, which play a critical role in degrading intracellular proteins. Tracking the motion of lysosomes appears to be a very powerful tool in analyzing cancer cells and can help with developing anti-cancer drugs. Analyzing the morphology of organelles can shed a lot of light on cellular function, but the morphometry of organelles is not an easy task. That is why a paper by Garza-Lopez et al. [4] appears to be a breakthrough approach in accomplishing this complicated assignment. They successfully worked out a standardized protocol utilizing the sophisticated image processing software Amira for accurate quantification of the 3D morphology of mitochondria and components of endoplasmic reticulum. This well-thought-out protocol will definitely attract a large number of researchers to analyze the morphology of other intracellular organelles.

Currently there are many image-processing software packages on the market, and end-users prefer the ones that are easy to use. A paper by Vieyres et al. [5] elaborates on complementary ImageJ plugins, PicPreview, and PicSummary, which simplify the manipulation of microscopy images and allow for the overview of all collected images in a few mouse clicks. These plugins are particularly helpful when preparing microscopy images for reports and publications.

The detrimental effects of chronic alcohol exposure are well known. In particular, there is a lot of interest in unraveling the molecular mechanisms underlying alcohol’s effects on liver hepatocytes. However, creating reliable in vitro conditions of liver cell exposure to alcohol is quite challenging due to the fact that ethanol evaporates very quickly when added directly to culture media. To overcome this problem, Kim et al. [6] developed a glass capillary system that steadily delivers and releases ethanol to cultured cells for several days, resembling a chronic alcohol exposure. This allows for the precise regulation of alcohol release conditions over a long period and analysis of the long-term effects of alcohol at a single-cell level.

Optimal cell culture conditions are of critical importance for maintaining cell viability and their proliferation capacity. Fetal Calf Serum (FCS) is a widely used component added to culture media. Unfortunately, FCS has disadvantages including the presence of endotoxin and infectious substances as well as ethical concerns about animal welfare because FCS is sourced from unborn calves at the slaughterhouse. Wanes et al. [7] replaced FCS with human platelet lysate (PL) to culture Caco-2 cells and did not find adverse effects of PL: Cell viability and differentiation were slightly improved, and the expression of vital biomarkers was not affected. This is a very important finding that may help eliminate the use of the controversial FCS as a cell culture additive. It will be interesting to see if using PL can be also used to improve cell culture of primary fibroblasts from leporid species: Abade dos Santos et al. described a simple, inexpensive technique [8] to generate the primary cell culture of fibroblasts from leporid species (rabbits and hares), which are widely used for biomedical research. Their novel technique allows the achievement of high viability and homogeneity of fibroblasts.

Another detailed step-by-step protocol has been presented by Nur Zahirah binte M. Yusoff et al. [9] focused on isolation, propagation, and cryopreservation of human Corneal Stromal Keratocytes (CSK) that can be used for corneal tissue engineering. Impaired vision caused by corneal opacification is a major medical problem that is one of the major causes of blindness in the world [10]. Developing protocols for obtaining human CSK that can be used for corneal transplantation is of critical importance to cure this eye disease.

The isolation and enrichment of cells with a particular phenotype is a routine procedure used by a large number of academic and biotech/pharma laboratories. One of the biggest challenges is obtaining a homogenous population of cells with a minimal number of irrelevant contaminating cells. Weiss et al. conducted a study to compare three positive selection techniques to purify human CD3 cells [11] using Magnetic Activated Cell Sorting (MACS^®^), Traceless Affinity Cell Selection (TACS^®^) and REAlease^®^, which utilizes low-affinity fragments rather than whole IgG molecules. Their report concludes that if there is a need to separate cells from the whole blood, then TACS^®^ and MACS^®^ are the preferred protocols, whereas TACS^®^ and REAlease^®^ are better suited for situations when the pre-activation and labeling of cells is required. Interestingly, none of three protocols had any advantage with regard to the function of cells and their proliferation. This comparative study not only provides food for thought but can also serve as a troubleshooting guide for laboratory personnel involved with optimization of cell separation techniques.

Microfluidic devices entered the cell biology field three decades ago [12], and their applications for cancer research have continued to grow ever since. Advantages of microfluidics include portability, high throughput, sensitivity, and low cost. Microfluidic chips use small sample sizes and allow for fast readout. A review by Liu et al. [13] provides a wealth of information on the application of microfluidics for analyzing circulating tumor cells (CTC) to investigate their reaction to specific signals in vitro in order to develop anti-cancer therapies. The use of microfluidics usually generates large datasets that have to be thoroughly analyzed by the operator, which can be an incredibly time-consuming operation. It appears that machine learning (ML) can be of great help with classification, prediction, and data extraction. ML application for microfluidics-centric cancer research is also reviewed in depth in this report, which makes it a great resource for both young investigators and cancer research experts.

No one argues against the benefits of physical exercise, which improves our strength, reduces the risk of cardiovascular disease, and has a strong positive impact on our mental health. However, what are the mechanisms underlying such benefits? Zhu et al. [14] addressed this question by using a mouse model of progressive resistance exercise (PRE) that resembles a physical human training regimen. They have reported that their mouse model is highly translatable to human PRE and can shed light on the mechanisms underlying the increase in muscle mass and long-term adaptations such as fiber hypertrophy and exercise-induced remodeling in skeletal muscle. Availability of such a mouse model opens the door to finding more efficient ways to improve muscle function via physical exercises.

The Guest Editor and authors believe that this Special Issue of *Cells* has a collection of diverse cell biology techniques and see it as a valuable addition to current protocols that can help improve cell biology skills by expanding our knowledge and inspiring our curiosity.

## References

[1] Campioni G., Pasquale V., Busti S., Ducci G., Sacco E., Vanoni M. (2022). An Optimized Workflow for the Analysis of Metabolic Fluxes in Cancer Spheroids Using Seahorse Technology. Cells.

[2] Born-Torrijos A., Kosakyan A., Patra S., Pimentel-Santos J., Panicucci B., Chan J., Korytář T., Holzer A. (2022). Method for Isolation of Myxozoan Proliferative Stages from Fish at High Yield and Purity: An Essential Prerequisite for In Vitro, In Vivo and Genomics-Based Research Developments. Cells.

[3] Polev K., Kolygina D., Kandere-Grzybowska K., Grzybowski B. (2022). Large-Scale, Wavelet-Based Analysis of Lysosomal Trajectories and Co-Movements of Lysosomes with Nanoparticle Cargos. Cells.

[4] Garza-Lopez E., Vue Z., Katti P., Neikirk K., Biete M., Lam J., Beasley H., Marshall A., Rodman T., Christensen T. (2022). Protocols for Generating Surfaces and Measuring 3D Organelle Morphology Using Amira. Cells.

[5] Vieyres G. (2021). PicPreview and PicSummary: Two Timesaving Plugins for the Fluorescence Microscopist. Cells.

[6] Kim W., Jeong H., Kim S., Choi C., Lee K. (2021). Chronic Alcohol Exposure of Cells Using Controlled Alcohol-Releasing Capillaries. Cells.

[7] Wanes D., Naim H., Dengler F. (2021). Proliferation and Differentiation of Intestinal Caco-2 Cells Are Maintained in Culture with Human Platelet Lysate Instead of Fetal Calf Serum. Cells.

[8] Abade dos Santos F., Carvalho C., Almeida I., Fagulha T., Rammos F., Barros S., Henriques M., Luís T., Duarte M. (2021). Simple Method for Establishing Primary Leporidae Skin Fibroblast Cultures. Cells.

[9] Binte M., Yusoff N., Riau A., Yam G., binte Halim N., Mehta J. (2022). Isolation and Propagation of Human Corneal Stromal Keratocytes for Tissue Engineering and Cell Therapy. Cells.

[10] Whitcher J.P., Srinivasan M., Upadhyay M.P. (2001). Corneal blindness: A global perspective. Bull. World Health Organ..

[11] Weiss R., Gerdes W., Berthold R., Sack U., Koehl U., Hauschildt S., Grahnert A. (2021). Comparison of Three CD3-Specific Separation Methods Leading to Labeled and Label-Free T Cells. Cells.

[12] Hong J.W., Quake S.R. (2003). Integrated nanoliter systems. Nat. Biotechnol..

[13] Liu Y., Li S., Liu Y. (2022). Machine Learning-Driven Multiobjective Optimization: An Opportunity of Microfluidic Platforms Applied in Cancer Research. Cells.

[14] Zhu W., Hibbert J., Lin K., Steinert N., Lemens J., Jorgenson K., Newman S., Lamming D., Hornberger T. (2021). Weight Pulling: A Novel Mouse Model of Human Progressive Resistance Exercise. Cells.

